# Deletion of *Wntless* in myeloid cells exacerbates liver fibrosis and the ductular reaction in chronic liver injury

**DOI:** 10.1186/s13069-015-0036-7

**Published:** 2015-10-15

**Authors:** Katharine M. Irvine, Andrew D. Clouston, Victoria L. Gadd, Gregory C. Miller, Weng-Yew Wong, Michelle Melino, Muralidhara Rao Maradana, Kelli MacDonald, Richard A. Lang, Matthew J. Sweet, Antje Blumenthal, Elizabeth E. Powell

**Affiliations:** Centre for Liver Disease Research, School of Medicine, The University of Queensland, Translational Research Institute, 37 Kent St, Brisbane, 4102 Australia; QIMR Berghofer Medical Research Institute, Brisbane, Australia; The University of Queensland Diamantina Institute, Translational Research Institute, Brisbane, Australia; Visual Systems Group, Cincinnati Children’s Hospital Medical Center, Cincinnati, OH USA; Institute for Molecular Bioscience and the Centre for Inflammation and Disease Research, The University of Queensland, Brisbane, Australia; Australian Infectious Diseases Research Centre, The University of Queensland, Brisbane, Australia

**Keywords:** Liver fibrosis, Ductular reaction, Macrophages, Matrix remodelling, TAA, CDE

## Abstract

**Background:**

Macrophages play critical roles in liver regeneration, fibrosis development and resolution. They are among the first responders to liver injury and are implicated in orchestrating the fibrogenic response via multiple mechanisms. Macrophages are also intimately associated with the activated hepatic progenitor cell (HPC) niche or ductular reaction that develops in parallel with fibrosis. Among the many macrophage-derived mediators implicated in liver disease progression, a key role for macrophage-derived Wnt proteins in driving pro-regenerative HPC activation towards a hepatocellular fate has been suggested. Wnt proteins, in general, however, have been associated with both pro- and anti-fibrogenic activities in the liver and other organs. We investigated the role of macrophage-derived Wnt proteins in fibrogenesis and HPC activation in murine models of chronic liver disease by conditionally deleting *Wntless* expression, which encodes a chaperone essential for Wnt protein secretion, in LysM-Cre-expressing myeloid cells (LysM-Wls mice).

**Results:**

Fibrosis and HPC activation were exacerbated in LysM-Wls mice compared to littermate controls, in the absence of an apparent increase in myofibroblast activation or interstitial collagen mRNA expression, in both the TAA and CDE models of chronic liver disease. Increased *Epcam* mRNA levels paralleled the increased HPC activation and more mature ductular reactions, in LysM-Wls mice. Increased Epcam expression in LysM-Wls HPC was also observed, consistent with a more cholangiocytic phenotype. No differences in the mRNA expression levels of key pro-inflammatory and pro-fibrotic cytokines or the macrophage-derived HPC mitogen, *Tweak*, were observed. LysM-Wls mice exhibited increased expression of *Timp1*, encoding the key Mmp inhibitor Timp1 that blocks interstitial collagen degradation, and, in the TAA model, reduced expression of the anti-fibrotic matrix metalloproteinases, *Mmp12* and *Mmp13*, suggesting a role for macrophage-derived Wnt proteins in restraining fibrogenesis during ongoing liver injury.

**Conclusion:**

In summary, these data suggest that macrophage-derived Wnt proteins possess anti-fibrogenic potential in chronic liver disease, which may be able to be manipulated for therapeutic benefit.

**Electronic supplementary material:**

The online version of this article (doi:10.1186/s13069-015-0036-7) contains supplementary material, which is available to authorized users.

## Background

Regardless of their aetiology, chronic liver diseases (CLDs) share a common pathological mechanism, liver-injury stimulated fibrosis, which, if progressive, can lead to cirrhosis, liver failure, and cancer. The inflammatory response to chronic injury, mediated by multiple parenchymal and non-parenchymal cells, plays critical roles in fibrogenesis. Like inflammation, fibrosis is an adaptive response to liver injury that, when appropriately regulated, facilitates tissue repair. How these protective responses become maladaptive and the interplay between fibrogenesis and repair, in the context of chronic injury and inflammation, is not well understood. Clinical observations and experimental models suggest that liver fibrosis is a dynamic, bidirectional process with the capacity for recovery even at advanced stages [1]. With no therapies available to prevent or treat liver fibrosis, better understanding of the mechanisms of fibrosis and repair and the role of inflammatory cells and mediators is crucial to developing new therapeutic approaches.

In a healthy liver, injured parenchymal cells can be regenerated through division of mature epithelial cells (hepatocytes and bile duct-forming cholangiocytes). Following severe or chronic injury, hepatocyte regeneration is impaired and an alternative regenerative pathway involving hepatic progenitor cells (HPCs) can be activated, which is associated with the development of ductular reactions (DRs) and progressive fibrosis, both in humans and mouse models of CLD [2–4]. HPCs are thought to be bipotential, capable of proliferation and differentiation into hepatocytes, to replace injured cells or along the ‘ductular’ cholangiocyte lineage. Although capable of differentiation into hepatocytes and cholangiocytes under clonogenic conditions in vitro, the extent to which HPCs directly replace epithelial cells to regenerate the liver is controversial [5–7]. The contribution of HPCs to liver regeneration in mouse models likely depends on the extent to which epithelial self-renewal is impaired. Indeed, it was recently demonstrated that HPC activation in the setting of near total hepatocyte death or senescence was necessary for survival [7]. DRs correlate with the extent of liver fibrosis and may be postulated to represent a failed attempt to regenerate the liver, but the causal relationship between DRs and fibrosis remains to be established. HPCs are intimately associated with fibrogenic activity, however, as they exist in a niche composed of myofibroblasts, macrophages and other leukocytes and extracellular matrix components. Both cellular and matrix components of the niche shape HPC activation and fate, and HPCs, in turn, influence myofibroblasts and inflammatory cells.

Macrophages play critical roles in liver regeneration, fibrosis development and resolution and are prominent features of the fibrotic scar and DR niche [8–11]. Macrophages have been shown to play a dichotomous role in the context of liver fibrosis, as they play a pro-fibrogenic role during the development of fibrosis whereas macrophage expression of matrix metalloproteinases and pro-angiogenic factors crucially contributes to the resolution of fibrosis during recovery from liver injury [10, 12] and potentially even during ongoing injury [13]. Macrophage-derived factors, including Wnt proteins, are also directly implicated in controlling HPC proliferation, differentiation and invasion into the parenchyma [14–16]. Macrophage ablation reduced DR proliferation and expansion in several models of CLD [10, 16] and, strikingly, injection of macrophages caused transient TWEAK-dependent HPC activation in healthy mice [15].

HPC activation during injury appears to recapitulate liver epithelial cell development from hepatoblasts during embryogenesis and involves reactivation of developmental cell signalling pathways, including Wnt, Notch and Hedgehog [17]. Wnt signalling drives hepatocellular differentiation during development [18], and a similar function for Wnt proteins (‘Wnts’) during chronic liver injury has been suggested [14]. In contrast, macrophage-derived Wnt7b was recently shown to drive cholangiocarcinoma growth [19], highlighting the complex role of Wnt signalling in chronic liver disease. Inflammatory cytokines (e.g. IL-6, TNF, LTβ, IFNγ) [20–23], injury-induced growth factors (e.g. TWEAK [24]) and extracellular matrix components (e.g. collagen, laminin and fibronectin [25, 26]) are also implicated in regulating HPC proliferation and fate.

Wnts comprise a family of 19 secreted glycoproteins that activate target cells via β-catenin and β-catenin-independent pathways to regulate cell proliferation, differentiation and migration during development and homeostasis, including in multiple adult stem cell niches [18]. The pleiotropic targets and biological effects of Wnt signalling in the liver are not well understood. A loss-of-function mutation in the LRP6 gene, encoding a Wnt co-receptor, was recently associated with human non-alcoholic fatty liver disease [27]. Introduction of this mutation in mice caused steatohepatitis via a β-catenin-independent pathway, which was rescued by therapeutic administration of Wnt3a [27]. β-catenin signalling is implicated in liver repair and fibrogenesis, but the extent to which this is Wnt-dependent is not clear, as β-catenin stabilisation also occurs downstream of non-Wnt growth factors, such as hepatocyte growth factor [28]. In patients and mice with acute liver failure, β-catenin activation correlated with increased liver regeneration and (in patients) precluded the need for transplantation [29]. Multiple studies in rodent models of CLD have also suggested a role for β-catenin signalling in chronic liver injury, including driving HPC proliferation and differentiation [30–34]. By contrast, Wnt/β-catenin signalling in fibroblasts was reported to be pro-fibrotic in systemic sclerosis, skin, lung, liver and kidney disease [35–38]. The cellular source and type of Wnt proteins expressed in response to injury and their target cells may determine whether tissue regeneration or fibrosis occurs. Macrophage- but not epithelial-derived Wnts contributed to liver regeneration after partial hepatectomy [39]; however, the role of macrophage-derived Wnts in chronic liver disease, in particular in the HPC niche, is unknown. In the current study, we investigated the contribution of macrophage-derived Wnts to HPC activation and fibrogenesis during chronic liver disease. To do so, we examined the phenotype of mice with myeloid-targeted deletion of Wntless (Wls), encoding the chaperone Wls required for Wnt secretion [40]. Mice with Wls-deficient macrophages exhibited enhanced ductular reactions and fibrosis in two murine models of chronic liver disease. Our data are consistent with roles for macrophage-derived Wnts in HPC maturation and matrix remodelling to restrain net collagen deposition during chronic liver disease progression.

## Results

### Myeloid-specific Wls knockout does not affect liver macrophage abundance or localisation

**Fig. 1:**
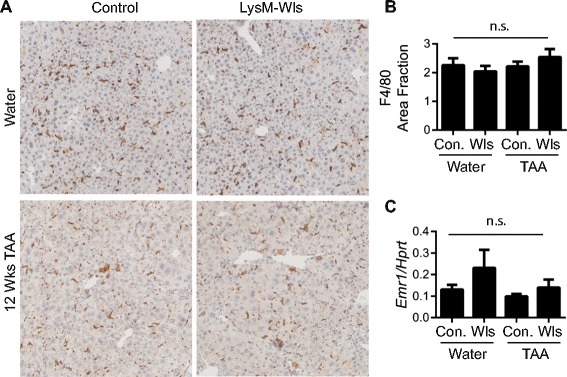
LysM-Wls depletion does not affect macrophage abundance and localisation. F4/80+ liver macrophage localisation (representative stain, 5× original magnification) (**a**). Quantification of F4/80+ staining (**b**) and whole liver *Emr1* (encoding the F4/80 antigen) expression (**c**) in control and LysM-Wls mice treated with water or TAA for 12 weeks. Data represent mean + SEM from 5 to 12 mice from 4 independent experiments. *n.s.* non-significant

To investigate the contribution of macrophage-derived Wnt proteins to inflammation, HPC activation and fibrosis, we generated mice deficient in *Wls* expression in myeloid cells by crossing Wls-loxp mice [40] with LysM-Cre mice [41] for 4–10 generations. Wls knockout in macrophages was confirmed by qPCR for *Wls* in bone marrow-derived macrophages (Additional file 1: Figure S1A). LysM-Wls (Cre+) mice and littermate controls (Cre-) were administered thioacetamide (TAA, 300 mg/L) in drinking water for 12 weeks as a model of chronic liver injury. Reduced *Wls* expression in F4/80+ liver cells sorted by flow cytometry was confirmed by qPCR (Additional file 1: Figure S1B–E). By contrast, *Wls* expression in Epcam+ cells, sorted by flow cytometry, was comparable between untreated Cre+ and Cre− animals, confirming the specificity of targeting (Additional file 1: Figure S1F). Consistent with a recent report [39], LysM-Wls mice were viable and livers were histologically normal. Wls deficiency did not affect the number or localisation of liver macrophages in control or TAA-treated mice, as assessed by immunohistochemistry and image analysis for F4/80 and qPCR for *Emr1* (encoding the F4/80 antigen) in whole liver (Fig. 1). To determine which Wnt proteins are expressed by liver macrophages, we conducted a PCR for a panel of Wnt ligands. *Wnt3a*, *Wnt5a*, *Wnt7a*, *Wnt7b* and *Wnt1* mRNAs were below the limit of detection in F480+ cells from control or TAA-treated mice; however, *Wnt4* and *Wnt6* were robustly detected but not significantly altered by TAA treatment or genotype (Additional file 1: Figure S1G–H).

### Reduction in Wnt/β-catenin signalling during liver injury in mice with Wls-deficient macrophages

**Fig. 2:**
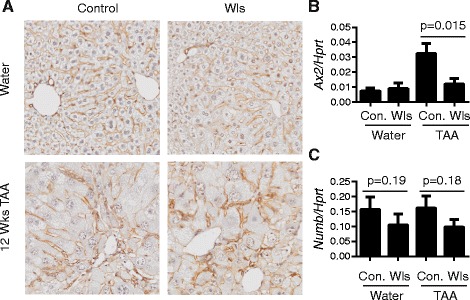
Reduced *Wls* expression in macrophages from LysM-Wls mice is associated with reduced β-catenin target gene expression. **a** β-Catenin staining (representative, 10× original magnification) and whole liver expression of **b** *Axin2* and **c** *Numb* in control and LysM-Wls mice treated with water or TAA for 12 weeks. Data represent mean+/−SEM of 5–12 mice from 4 independent experiments

In healthy livers, β-catenin localised to hepatocyte membranes and bile duct epithelia, whereas after 12 weeks TAA treatment, β-catenin stained hepatocyte membranes and some nuclei, as well as HPC, in both LysM-Wls mice and littermate controls (Fig. 2a). Whilst no clear difference in β-catenin expression or localisation in injured mice was discernible, expression of the Wnt/β-catenin target gene, A*xin2*, was elevated in livers from TAA-treated control mice but not LysM-Wls mice (Fig. 2b), consistent with a reduction in Wnt/β-catenin signalling in LysM-Wls mice during liver injury. Whole liver expression of the Wnt/β-catenin target genes *Ccnd2* and *Numb,* which has previously been suggested to contribute to HPC hepatocellular fate determination [14], was not significantly different between genotypes, although there was a trend towards reduced *Numb* expression in LysM-Wls mice (Fig. 2c and data not shown).

### Wls depletion in macrophages exacerbates TAA-induced hepatic progenitor cell activation and fibrosis

**Fig. 3:**
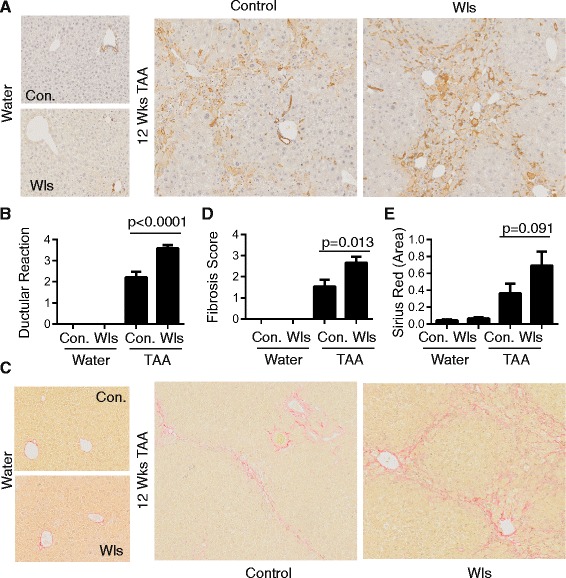
Increased ductular reaction and fibrosis in LysM-Wls mice. **a** Representative CKWSS staining and **b** ductular reaction grade in livers from 12-week water- and TAA-treated control and LysM-Wls mice. **c** Representative Picro-Sirius Red staining, **d** fibrosis score and **e** collagen proportionate area in livers from water- and 12-week TAA-treated control and LysM-Wls mice. Data represent mean + SEM of 8–17 mice from 4 independent experiments

**Fig. 4:**
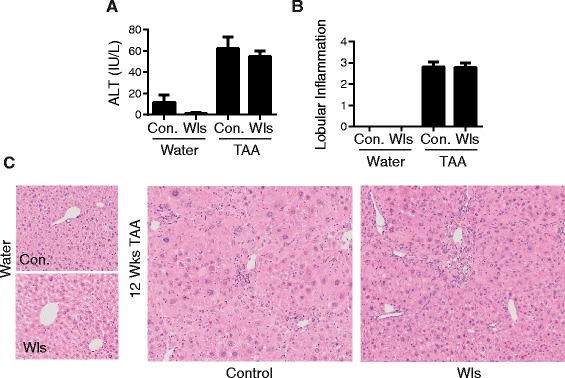
Comparable injury and inflammation in LysM-Wls and control mice. **a** Serum ALT levels in water- and 12-week TAA-treated control and LysM-Wls mice. Data represent mean+/−SEM of 8–10 mice from 2 independent experiments. **b** Lobular inflammation score and **c** representative H&E staining, in water- and 12-week TAA-treated control and LysM-Wls mice. Data represent mean + SEM of 8–17 mice from 4 experiments

**Fig. 5:**
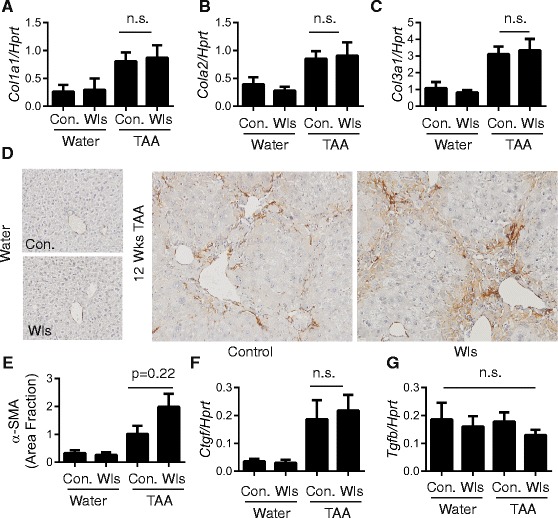
Comparable myofibroblast activation in LysM-Wls and control mice. Whole liver expression of **a** *Col1a1*, **b** *Col1a2* and **c** *Col3a1* in water- and 12-week TAA-treated control and LysM-Wls mice. **d** Representative SMA staining and **e** quantification of SMA staining (mean + SEM, *n* = 5 per group) in livers from water- and 12-week TAA-treated control and LysM-Wls mice. Whole liver expression of **f** *Ctgf* and **g** *Tgfb* in water- and 12-week TAA-treated control and LysM-Wls mice. Data represent mean + SEM of 8–17 mice from 4 independent experiments. *n.s.* not significant

After 12 weeks of TAA treatment, LysM-Wls mice exhibited increased HPC activation, frequently associated with mature ductular reactions, which were not observed in littermate controls (pan-keratin staining, Fig. 3a, b). This was associated with increased fibrosis (Picro-Sirius red staining, Fig. 3c–e). Female mice were used in this study because we observed that TAA-induced HPC activation and fibrosis were more severe in females than males, however Wls deficiency in macrophages similarly exacerbated the DR and fibrosis in male mice (Additional file 2: Figure S2). The increased DR and fibrosis was not explained by increased susceptibility to TAA-induced injury, as liver injury was comparable in LysM-Wls mice and littermate controls, as assessed by serum ALT and liver histology (Fig. 4a–c). The expression of the key pro-inflammatory cytokines *Tnf* and *Il6* that are produced by macrophages and other cell types in response to liver injury and play critical roles in liver inflammation, fibrosis and HPC activation [20, 22, 42] was not significantly different between LysM-Wls and littermate controls (data not shown). Despite the increase in fibrosis in LysM-Wls mice, mRNA levels of the key interstitial collagens contributing to liver fibrosis [43], *Col1a1*, *Col1a2* and *Col3a1*, were comparable between LysM-Wls and littermate controls (Fig. 5a–c). Although mRNA expression of *Sma*, which encodes smooth muscle actin, was comparable between genotypes (data not shown), there was a trend towards increased Sma protein expression in LysM-Wls mice (Fig. 5d, e), suggesting an increase in myofibroblast activation in these mice. Nevertheless, mRNA expression of the pro-fibrogenic cytokines *Tgfb* and *Ctgf* [44] that play key roles in myofibroblast activation did not differ between genotypes (Fig. 5f, g).

### Hepatic progenitor cells express an immature profile in mice with Wls-deficient macrophages

**Fig. 6:**
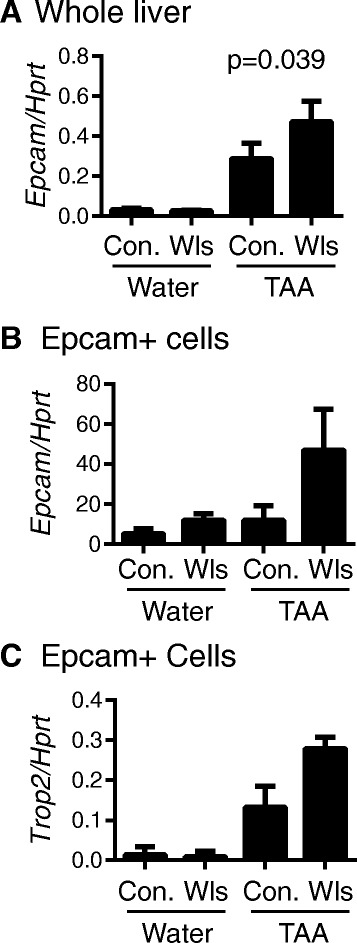
Increased Epcam expression in Epcam+ cells from LysM-Wls mice. **a** Whole liver expression of *Epcam* in water- and 12-week TAA-treated control and LysM-Wls mice (mean + SEM of 8–17 mice from 4 independent experiments). Expression of **b** Epcam and **c** Trop2 in flow cytometry-sorted Epcam+ cells (pooled from 3 to 6 mice) from water- and 12-week TAA-treated control and LysM-Wls mice (mean + range of 2 independent experiments)

Paralleling the ductular reaction, Epcam (*Tacstd1*) liver mRNA levels were elevated in LysM-Wls mice compared to littermate controls (Fig. 6a), whereas the expression of the biliary keratin, *Krt19*, did not differ. Epcam is a cell surface glycoprotein expressed on tissue stem cells with regenerative capacity and is normally downregulated upon terminal differentiation [45]. To investigate the effects of macrophage-derived Wnts on HPC gene expression, we sorted Epcam+ cells from the liver non-parenchymal fraction to enrich for HPC. Expression of Epcam itself was increased in Epcam+ cells from TAA-treated LysM-Wls mice compared to littermate controls (Fig. 6b). *Trop2* (*Tacstd2*), which encodes an Epcam-related protein reported to be specifically induced in HPC upon liver injury [46], was elevated in TAA-treated control mice and hyper-induced in LysM-Wls mice (Fig. 6c). We investigated the expression of the Notch target gene *Hey1* and the Wnt/β-catenin target genes *Axin2* and *Numb* in Epcam+ cells, as the Notch and Wnt/β-catenin pathways have previously been suggested to have opposing roles in determining HPC fate [14, 17], to provide evidence for direct effect of macrophage-derived Wnts on HPC, however these genes were only marginally detected in sorted cells (data not shown).

### Macrophage metalloproteinase expression is reduced in TAA-treated LysM-Wls mice

**Fig. 7:**
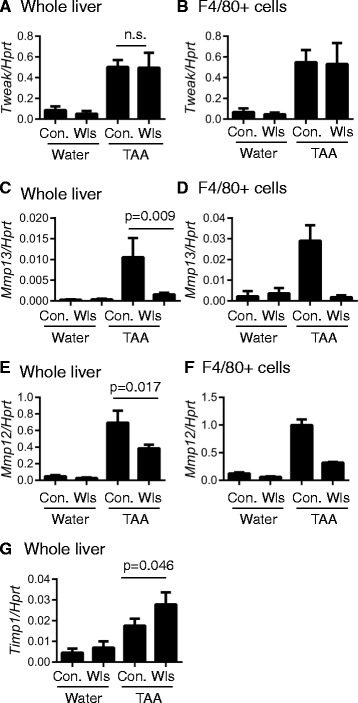
Reduced macrophage matrix metalloproteinase expression in LysM-Wls mice. *Tweak* expression in **a** whole liver and **b** flow cytometry-sorted macrophages. *Mmp13* expression in **c** whole liver and **d** flow cytometry-sorted macrophages, *Mmp12* expression in **e** whole liver and **f** flow cytometry-sorted macrophages and **g** whole liver expression of *Timp1* in water- and 12-week TAA-treated control and LysM-Wls mice. Data represent mean + SEM of 8–17 mice (whole liver) or mean + range of 2 independent experiments (sorted macrophages pooled from 3 to 6 mice in each experiment)

Macrophage production of the HPC mitogen, Tweak*,* has been implicated in driving HPC proliferation [15, 24]. Although TAA inducible, *Tweak* expression did not differ in whole liver or flow cytometry-sorted F4/80+ cells from control and LysM-Wls mice (Fig. 7a, b). As in whole liver, expression of the pro-inflammatory cytokines, *Il6* and *Tnf*, and the pro-fibrotic cytokine, *Tgfb*, did not differ between LysM-Wls and control macrophages (data not shown). Since macrophages play a key role in matrix remodelling, we also examined the expression of key matrix remodelling factors implicated in liver fibrosis and repair [47]. We found myeloid deletion of Wls impaired injury-mediated upregulation of *Mmp13* (encoding the key interstitial collagenase in rodents, Mmp13) and *Mmp12* (encoding the elastase Mmp12) mRNAs in whole liver and purified F4/80+ cells (Fig. 7c–f). *Mmp2* and *Mmp8* were induced in TAA-injured livers but not differentially expressed in Wls-deficient and control mice, whereas no alteration in liver or macrophage *Mmp9* expression was observed (data not shown). Furthermore, in TAA-treated LysM-Wls mice, the expression of *Timp1*, encoding the key Mmp inhibitor Timp1 that blocks interstitial collagen degradation [1], was higher compared to littermate controls (Fig. 7g). These data are consistent with the hypothesis that fibrolysis, as well as fibrogenesis, is dynamically regulated during TAA-mediated liver injury and suggest that macrophage-derived Wnt proteins promote matrix remodelling, via Mmp13 and Mmp12, potentially restraining fibrosis.

### Wls depletion in macrophages exacerbates CDE-induced hepatic progenitor cell activation and fibrosis

To determine whether macrophage-derived Wnts contribute to the regulation of HPC activation and fibrogenesis in multiple contexts of liver injury, we employed an alternative model of liver injury and fibrosis. The choline-deficient ethionine-supplemented (CDE) diet induces a robust DR but a different pattern of fibrosis to TAA [48]. Liver injury and fibrosis were also more severe in female mice than male mice in this model (data not shown). Similar to the TAA model, HPC activation and fibrosis were exacerbated in CDE-treated LysM-Wls mice compared to littermate controls, in the absence of alterations in whole liver *Col1a1*, *Col1a2*, *Col3a1*, *Sma* and *Il6* expression between LysM-Wls mice and littermate controls (Additional file 3: Figure S3A–E and data not shown). Epcam expression was elevated in CDE-treated LysM-Wls mice, in whole liver and Epcam+ cells (Additional file 3: Figure S3F, G), suggesting similar alterations in HPC phenotype in both models. As in the TAA model, Timp1 mRNA expression was increased in CDE-treated LysM-Wls mice compared to littermate controls (Additional file 3: Figure S3I); however, Mmp13 was not detectable in the majority of CDE-treated mice and there was no difference in Mmp12, Mmp8, Mmp2 or Mmp9 expression (data not shown). Wnt4 and Wnt6 were expressed in macrophages from CDE-treated mice, regardless of genotype (data not shown).

## Discussion

Macrophages contribute to the dynamic interplay between liver inflammation, fibrosis and regeneration via multiple mechanisms, at different locations and different disease stages. Inflammatory, bone marrow-derived monocytes infiltrate the liver in response to injury, where they are dynamically reprogrammed, depending on the nature and chronicity of the injury [10, 49–52]. Macrophages are among the earliest responders to liver injury, having roles in phagocytosis of apoptotic and necrotic cells and releasing soluble mediators, such as TNF and IL-6, that orchestrate inflammatory and ultimately pro-fibrogenic responses to chronic injury. Macrophages are also prominent components of the hepatic progenitor cell niche, which is closely associated with liver fibrogenesis [2, 8, 9] and repair; although the fate relationships among hepatocytes, cholangiocytes and HPC and the contribution of HPC to liver fibrosis and regeneration are controversial [5–7, 53–55]; and the factors determining their activation and differentiation are not well understood. Wnt proteins, potentially derived from macrophages, have been suggested to promote liver regeneration by driving HPC hepatocellular differentiation [14, 17, 32], which could theoretically ameliorate liver fibrosis by promoting regeneration. Wnt proteins, however, constitute a large family of proteins that have been variously attributed pro- and anti-fibrogenic properties [35–38] and which likely have multiple potential target cells and mechanisms of action during chronic liver disease. We investigated the role of macrophage-derived Wnt proteins in fibrogenesis in two models of chronic liver disease and demonstrate that ablating Wls expression in myeloid cells exacerbated HPC activation and fibrosis.

Although the role of β-catenin in liver homeostasis and injury has been extensively studied [18], the contribution and sources of specific β-catenin-dependent Wnt proteins are little studied, and β-catenin-independent Wnt signalling is even less studied [27, 32, 39]. β-catenin is essential for hepatocyte-mediated liver regeneration after partial hepatectomy, and it was recently shown that macrophage- but not epithelium-derived Wnt proteins contribute to β-catenin activation in this setting [39]. A novel, β-catenin-dependent population of self-renewing hepatocytes maintained by endothelial-derived Wnts was also recently identified, although the role of these cells in liver injury has not yet been investigated [56]. In humans with acute liver failure, nuclear β-catenin localisation in hepatocytes correlated with patient survival and reduced requirement for liver transplant [29], consistent with a pro-regenerative role for β-catenin, although Wnt proteins were not directly implicated in this study. Interestingly, β-catenin depletion from mature hepatocytes in healthy mice caused HPC expansion, and hepatocytes were gradually replaced by β-catenin-positive HPC [57]. β-catenin has also been implicated in HPC-mediated liver regeneration in murine chronic liver disease [30, 33], and hepatocyte-derived Wnt1 promoted HPC differentiation in rats, following partial hepatectomy and 2-acetylaminofluorene (AAF) treatment [32]. Our data demonstrating increased fibrosis and associated with more extensive ductular reactions and a more cholangiocytic HPC phenotype (increased Epcam and Trop2 expression) in LysM-Wls mice are consistent with a role for Wnt proteins in protecting from fibrosis by promoting HPC hepatocellular differentiation. However, we did not observe any difference in β-catenin localisation in livers from LysM-Wls and control mice. Whilst the β-catenin target gene *Axin2* was reduced in whole liver in LysM-Wls mice, it was below the limit of detection in Epcam+ cells (which include HPC), so we could not ascertain whether this pathway was differentially activated in these cells in the absence of macrophage-derived Wnts. It is possible that cells with activated β-catenin signalling are not represented among the sorted population due to maturation-associated Epcam downregulation [45]. Alternatively, rather than representing a block in hepatocellular differentiation, the expanded DR observed in LysM-Wls mice could result from a pro-proliferative drive, via the activation of Hedgehog or other growth factors [58, 59], the persistence of collagen or altered matrix composition [11], or from hepatocyte metaplasia, rather than HPC activation [54].

In addition to their potential direct (or indirect) effects on HPC, macrophage-derived Wnts could influence HPC activation and fibrosis via additional target cells and mechanisms. Wnt/β-Catenin signalling, for example, has been implicated in promoting stellate cell quiescence [60]; but we did not observe any difference in SMA expression or key cytokine regulators of myofibroblast activation, in LysM-Wls and control mice. In both the TAA and CDE models, increased hepatic collagen deposition in LysM-Wls mice occurred in the absence of increased expression of the key interstitial collagens, *Col1a1*, *Col2a1 and Col3a1*. In contrast to a typical pro-fibrogenic signature, with evidence of increased myofibroblast activation and collagen production, Timp1 mRNA, encoding a key hepatic Mmp inhibitor that prevents remodelling of the fibrotic scar [61, 62], was increased in LysM-Wls mice compared to littermate controls in both the TAA and CDE models. Moreover, in the TAA model, macrophage Mmp13 and Mmp12 expression was reduced in LysM-Wls mice. These data suggest the hypothesis that macrophage-derived Wnts promote fibrolysis, which dynamically counter-balances collagen accumulation, even during active fibrogenesis. The contribution of matrix remodelling to fibrogenesis *during* ongoing liver injury is not well understood, but studies altering the Timp to Mmp balance have consistently confirmed the potent influence of this ratio on the development and resolution of liver fibrosis [1]. Macrophages clearly play an essential role in the resolution of established fibrosis, after cessation of liver injury [10, 51, 52]. This involves reprogramming recruited monocytes/macrophages to a pro-resolution phenotype, including increased phagocytic activity and increased Mmp12 and Mmp13 expression, in conjunction with loss of liver Timp1 mRNA and protein expression [52]. Interestingly, administration of bone marrow-derived macrophages during ongoing toxic injury was sufficient to ameliorate fibrosis, and this was associated with increased Mmp13 expression [13]. Similarly, Barnes and colleagues recently reported a reduced rate of collagen degradation, associated with reduced recruitment of macrophages to the fibrotic scar, and reduced Mmp13 activity during CCL4-induced liver injury in mice lacking the chemokine MIF [63]. Mmp and Timp functions are not, however, limited to matrix proteolysis. Timp1 inhibits myofibroblast apoptosis [64], for example, whilst Mmp13 could contribute to fibrosis resolution via activation of latent hepatocyte growth factor and multiple downstream mechanisms [65–69]. Investigating the dynamic remodelling of multiple matrix components during chronic injury will be important to determine the functional relevance of the Timp1/Mmp balance in different models at different stages of disease progression.

## Conclusions

We have shown for the first time that the net effect of myeloid-derived Wnt proteins in chronic liver disease is to dampen HPC activation and fibrogenesis. Multiple Wnt proteins acting on different target cells may contribute to this phenotype, including direct effects on HPC fate, collagen-producing myofibroblasts and macrophages. Whilst Wnt induction, particularly leading to β-catenin activation, is not a viable therapeutic strategy in the setting of chronic liver disease, due to the risk of cancer development [70]; elucidation of the downstream pathways mediating Wnt proteins’ anti-fibrogenic effects may reveal novel, specific therapeutic targets. Most significantly, however, these data suggest that pro- and anti-fibrogenic macrophage functions co-exist during disease progression and support the notion that reprogramming macrophages or otherwise enhancing anti-fibrogenic pathways may be a viable therapeutic strategy to ameliorate fibrogenesis during chronic liver disease.

## Methods

### Transgenic mice and in vivo models

All animal experiments were performed with approval from the University of Queensland Animal Ethics Committee (MED/PAH/156/13/PAHRF/NHMRC, UQDI/571/12/NHMRC/AIDRCC). To conditionally delete Wls from macrophages, homozygous Wls^flox/flox^ mice [40] were crossed with LysM-Cre transgenic mice [41] for 4–10 generations. Offspring with the genotype Wls^flox/flox^; LysM-Cre-positive represents Wls macrophage knockouts, whilst Wls^flox/flox^; LysM-Cre-negative was used as controls. Six to 9-week-old mice were administered with 30 mg/L thioacetamide (TAA, Sigma) in drinking water for 12 weeks [71] or a modified choline-deficient ethionine-supplemented (CDE) diet for 6 weeks [48], to induce chronic liver injury and fibrosis. The modified CDE diet was optimised by Professor George Yeoh [48] (UWA, Australia) and was custom made by MP Biosciences (Santa Ana, USA). The diet consisted of 70 % choline-deficient diet (Cat#0296021410) and 30 % control (choline-sufficient) diet (Cat#0296041210). At the end of the treatment period, mice were euthanised and a blood sample taken for serum isolation. Livers were perfused with 10 ml of PBS via the portal vein in situ prior to tissue harvest to minimise blood contamination. Portions of the liver were fixed in 10 % neutral buffered formalin and embedded in paraffin or homogenised in TRI reagent and stored at −80 °C for RNA isolation. The remainder of the liver was used to isolate non-parenchymal cells. Serum ALT levels were measured using the MaxDiscovery Alanine Transaminase Color Endpoint Assay (Bioo Scientific, Austin, TX, USA).

### Non-parenchymal cell isolation

Non-parenchymal cells were isolated by finely dissociating the liver tissue using surgical scissors then passing the fragments through a stainless steel strainer with the addition of 14 ml of Hank’s Based Salt Solution (HBSS) containing 1 mg/ml type 4 collagenase (Worthington Biochemical corporation, Lakewood, NJ, USA) 1 μg/ml DNase I (Sigma), 100 U/ml penicillin and 100 μg/ml streptomycin. The cell suspension was filtered through a 70-μM filter (Becton Dickinson) and incubated at 37 °C for 5 min, prior to the addition of 10 mM EDTA and a further 5-min incubation period. The lysate was washed twice in cold PBS containing 2 % FBS (FACS buffer) and resuspended in 25 ml of 33 % Percoll in PBS (isotonic) and centrifuged at 600 × g for 15 min with no brake. The supernatant was discarded and the pellet washed twice and resuspended in FACS buffer. Freshly isolated non-parenchymal cells (~5 × 10^6^) per liver were stained with anti-F4/80-PE (clone BM8) and anti-Epcam-APC (clone G8.8) (Biolegend) antibodies for 30 min at room temperature, washed twice and resuspended in FACS buffer for sorting. PE- and APC-positive cells were sorted using an Astrios Moflo, achieving >90 % purity. Sorted cells were resuspended in TRI reagent (Sigma) for RNA purification.

### RNA purification and qPCR

RNA from whole liver (5 μg) or sorted cells (entire yield) was transcribed to cDNA using oligodT priming and Superscript III (Life Technologies). Quantitative real-time PCR (qPCR) for genes of interest was performed using SYBR green (ABI) on an HT9000 or VIA-VII cycler with default cycle settings. Relative expression was determined using *Hprt* as housekeeping gene and the delta-Ct method and expressed as Target/Hprt. Primer sequences used in this study were the following: Hprt-F: TGCTGGATTACATCAAAGCACTG, Hprt-R: CCCCTGTTGACTGGTCATTACAA; Ccnd1-F CACAACGCACTTTCTTTCCAGA Ccnd1-R CTTGACTCCAGAAGGGCTTCAA Numb-F CAATGAGTTGCCTTCCACTATGC Numb-R ATCTGGGAACACAAGGAGCTGA Mmp12-F TACCCCAAGCTGATTTCCACAC Mmp12-R GCTCCTTGGAAGATGTAGTAGTGTCTTT Mmp13-F ACAAAGATTATCCCCGCCTCAT Mmp13-R GGCCCATTGAAAAAGTAGATATAGCC Timp1-FAAGGGCTAAATTCATGGGTTCC Timp1-R ACAGCCTTGAATCCTTTTAGCATC Mmp9-F AGGGGCGTGTCTGGAGATTC Mmp9-R TCCAGGGCACACCAGAGAAC Tweak-F CGCTCTTAGTCTGGTCCTGGTTT Tweak-R CACCAGTCTCCTCTATGGGGGTA Tgfb-F GTGGCTGAACCAAGGAGACG Tgfb-R GGCTGATCCCGTTGATTTCC F480-F CTGTCTGCTCAACCGTCAGGTA F480-R AGAAGTCTGGGAATGGGAGCTAA E Epcam-F CGGGGATTGTTGTCCTGGTT Epcam-R GCACGGCTAGGCATTAAGCTC Trop2-F AGGAGCTGGGGGAGATGAGA Trop2-R CCAACCCATCTGGTCTGAGG Krt19-F CCTAGCCAAGATCCTGAGTGAGAT Krt19-R TGGGTGTTCAGCTCCTCAATC Ctgf-F GTCAAGCTGCCTGGGAAATG Ctgf-R AATGTGTCTTCCAGTCGGTAGGC Il6-F GCTGGTGACAACCACGGCCT Il6R GGCATAACGCACTAGGTTTGCCG Tnf-F AGGGGCCACCACGCTCTTCT Tnf-R CGGGGCAGCCTTGTCCCTTG Wls-F CAAATCGTTGCCTTTCTGGTG Wls-R TTGTCACACTTGTTAGGTCCC Wnt4-F AAGAGGAGACGTGCGAGAAA Wnt4-R CACCACCTTCCCAAAGACAG Wnt6-F TCAAGACTCTTTATGGATGCGC Wnt6-R ATGGCACTTACACTCGGTG Sma-F GATCCTGACTGAGCGTGGCTAT Sma-R CGTGGCCATCTCATTTTCAAAG mMmp2-F TTGCAGGAGACAAGTTCTGGAGATA mMmp2-R CACGACGGCATCCAGGTTAT mMmp8-F GGTTACCCCAAAAGCATACCAA mMmp8-R CACTGAAGAAGAGGAAGAAGGAGTC mCol1a2-F CACCCCAGCGAAGAACTCATAC mCol1a2-R CCCCTTCTACGTTGTATTCAAACTG mCol3a1-F TGGGATCAAATGAAGGCGAAT mCol3a1-R GCTCCATTCCCCAGTGTGTTTAG.

### Histology, immunohistochemistry and image analysis

H&E and Sirius red stains were performed and histological changes (inflammatory infiltrate and fibrosis) were assessed by two experienced hepatopathologists (GCM and ADC). Fibrosis stage was assessed using a modified METAVIR score as previously described [72]; briefly, stage 1: peri-portal or centrilobular fibrosis, stage 2: some septa, stage 3: many septa, stage 4: cirrhosis. Deparaffinised liver tissue sections were immunolabelled as described previously [73]. Antibodies used in this study were cytokeratin wide-spectrum screening (CKWSS, DAKO), F4/80 (Abcam), SMA (1A4, DAKO), β-catenin (Cell Signalling). HPC activation/ductular reactions were graded from CKWSS-stained sections according to the following schema: 1: CKWSS+ cells surrounding <50 % of portal vein, 2: CKWSS+ cells surrounding >50 % of portal vein; 3: CKWSS+ cells forming septa covering <50 % distance between two structures, 4: CKWSS+ cells forming septa covering >50 % distance between two structures. SMA and F4/80 image analysis were performed using ImageJ from ten captured images per section, each representing an area 310 × 250 μm in size. Sirius Red image analysis was performed using the positive pixel count algorithm in Aperio ImageScope from Aperio AT slide scans, from an area of 5–10 mm^2^ for each liver specimen. The algorithm was optimised to detect red pixels (hue value 0, hue width 0.1 and colour saturation threshold 0.22). All portal tracts and central veins greater than 100 μm in size were removed as they contain a large amount of collagen and therefore prevent quantification of fibrosis-associated collagen.

### Statistical analysis

Group comparisons were performed using the Mann–Whitney-*U* test. A two-tailed *p* value of 0.05 was considered statistically significant. All statistical analyses were performed using Prism Version 6 (GraphPad).

## Additional files

Additional file 1: Figure S1.
*Wls* depletion and *Wnt* expression in Wls-loxp × LysM-Cre mice. (A) RT-PCR quantification of Wls expression in bone marrow-derived macrophages from Wlsfl/fl and LysM-Cre+/− mice (n = 4–5, Mann–Whitney-*U* test). (B) Flow cytometry gating strategy for sorting F4/80+ and Epcam+ cells from liver non-parenchymal cells. Post-sort analysis of (C) F4/80+ and (D) Epcam+ cells. RT-PCR quantification of *Wls* expression in sorted (E) F4/80+ and (F) Epcam+ cells from control and 12-week TAA-treated mice. RT-PCR quantification of (G) *Wnt4* and (H) *Wnt6* expression in sorted F4/80+ cells from control and 12-week TAA-treated mice. Sorted cells were pooled from 3 to 6 mice from control and LysM-Wls mice. Data represent mean + range of 2 independent experiments. (EPS 1589 kb) Additional file 2: Figure S2. Increased ductular reaction and fibrosis in male LysM-Wls mice compared to littermate controls. (A) Ductular reaction grade, (B) fibrosis score and (C) whole liver *Col1a1* expression in livers from water- and 12-week TAA-treated control and LysM-Wls male mice. Data represent mean + SEM of 4–8 mice from 3 independent experiments. (EPS 478 kb) Additional file 3: Figure S3. Increased ductular reaction and fibrosis in LysM-Wls mice in the CDE model of chronic liver disease. Ductular reaction grade (A), fibrosis score (B) and whole liver expression of *Col1a1* (D), *Col3a1* (E), *Sma* (F), *Epcam* (G) and *Timp1* (I) in livers from LysM-Wls and control mice fed with a CDE diet for 6 weeks. Data represent mean + SEM from *n* = 3 mice from 1 experiment, representative of 3 independent experiments. (H) *Epcam* expression in flow cytometry-sorted Epcam+ cells (pooled from 3 mice) from LysM-Wls and control mice treated fed with a CDE diet for 6 weeks. Data represent mean + range of PCR duplicates, representative of 3 independent experiments. (EPS 509 kb)

## Abbreviations

CDE: Choline-deficient ethionine-supplemented
CLD: Chronic liver disease
DR: Ductular reaction
HPC: Hepatic progenitor cells
TAA: Thioacetamide
